# Changes in muscle-to-fat ratio are associated with lung function decline and airflow obstruction in the general population

**DOI:** 10.1186/s12931-024-03081-w

**Published:** 2024-12-26

**Authors:** Eunwoo Kim, Ah Young Leem, Ji Ye Jung, Young Sam Kim, Youngmok Park

**Affiliations:** 1 Yonsei University College of Medicine, Seoul, Republic of Korea; 2 Division of Pulmonary and Critical Care Medicine, Department of Internal Medicine, Severance Hospital, Yonsei University College of Medicine, Seoul, Republic of Korea; 3 Institute for Innovation in Digital Healthcare, Yonsei University, Seoul, Republic of Korea

**Keywords:** Lung function, Spirometry, Body composition, Sarcopenia, Obesity

## Abstract

**Background:**

The long-term relationship between body composition and lung function has not yet been fully demonstrated. We investigated the longitudinal association between muscle-to-fat (MF) ratio and lung function among middle-aged general population.

**Methods:**

Participants were enrolled from a community-based prospective cohort between 2005 and 2014. Lung function parameters (forced vital capacity [FVC], forced expiratory volume in 1 s [FEV_1_], and FEV_1_/FVC) and the MF ratio (total body muscle mass [kg]/fat mass [kg]) were assessed biannually via spirometry and bioelectrical impedance analysis, respectively.

**Results:**

We followed up 4,712 participants (age 53.9 ± 7.9 years, men 45.8%) for 8 years. With an increase in MF ratio of 1, in men, the FVC increased by 43.9 mL, FEV_1_ by 37.6 mL, and FEV_1_/FVC by 0.320%, while in non-smoking women, the FVC increased by 55.8 mL, FEV_1_ by 44.3 mL, and FEV_1_/FVC by 0.265% (all *P* < 0.001). The MF ratio-decreased group showed further annual deterioration in lung function than the MF ratio-increased group (men: FVC − 44.1 mL vs. -28.4 mL, FEV_1_ -55.8 mL vs. -39.7 mL, FEV_1_/FVC − 0.53% vs. -0.42%; non-smoking women: FVC − 34.2 mL vs. -30.3 mL, FEV_1_ -38.0 mL vs. -35.2 mL; all *P* < 0.001, except FEV_1_ in non-smoking women; *P* = 0.005). The odds ratio for the incidence of airflow obstruction according to the MF ratio was 0.77 (95% CI, 0.68–0.87) in men and 0.85 (95% CI, 0.74–0.97) in non-smoking women.

**Conclusions:**

Long-term changes in the MF ratio are related to lung function deterioration and incidence of airflow obstruction in middle-aged general population.

**Supplementary Information:**

The online version contains supplementary material available at 10.1186/s12931-024-03081-w.

## Introduction

Changes in body composition, especially in muscle and fat mass, are the most noticeable effects of aging [1, 2]. As a result of a complex etiology, loss of muscle mass and function, or sarcopenia, has increasing prevalence with age [3]. This geriatric change is commonly matched by increased body fat, defined as obesity. Obesity can independently cause sarcopenia because of the negative effects of adipose tissue-dependent metabolic disturbances such as oxidative stress and inflammation [4]. Muscle loss may also contribute to fat formation by lowering total energy expenditure [5, 6]. This vicious cycle of muscle loss and fat gain affects the whole body, becoming a significant risk factor for diverse diseases and frailty, which are especially harmful to lung function [7–9].

Previous studies, via various body composition parameters such as skeletal muscle index and fat-free mass, have shown a close association between lung function and muscle or fat mass. Most studies were cross-sectional, not reflecting changes in the participants over time [10–12]. However, it is important to show the longitudinal impact of body composition on lung function because fat and muscle mass changes are closely linked with aging [6]. Furthermore, most of the studies were based on patients with chronic obstructive pulmonary disease (COPD) or those with lung-associated diseases. Therefore, the influence of fat and muscle change on lung function in the general population remains unclear [11–15]. Thus, investigating the relationship in the general population who do not have apparent lung diseases will be necessary.

In this study, we explored the long-term relationship between lung function and muscle-to-fat (MF) ratio in middle-aged general population. Thereafter, we demonstrate the impact of the MF ratio on airflow obstruction.

## Methods

### Study participants

This study used data from the Korean Genome and Epidemiology Study (KoGES), a prospective community-based cohort. The cohort consists of 10,030 men and women aged 40–69 years old, living in Ansan (an urban community) and Anseong (a rural community). A follow-up study has been conducted there biannually from 2001 [16].

**Fig. 1:**
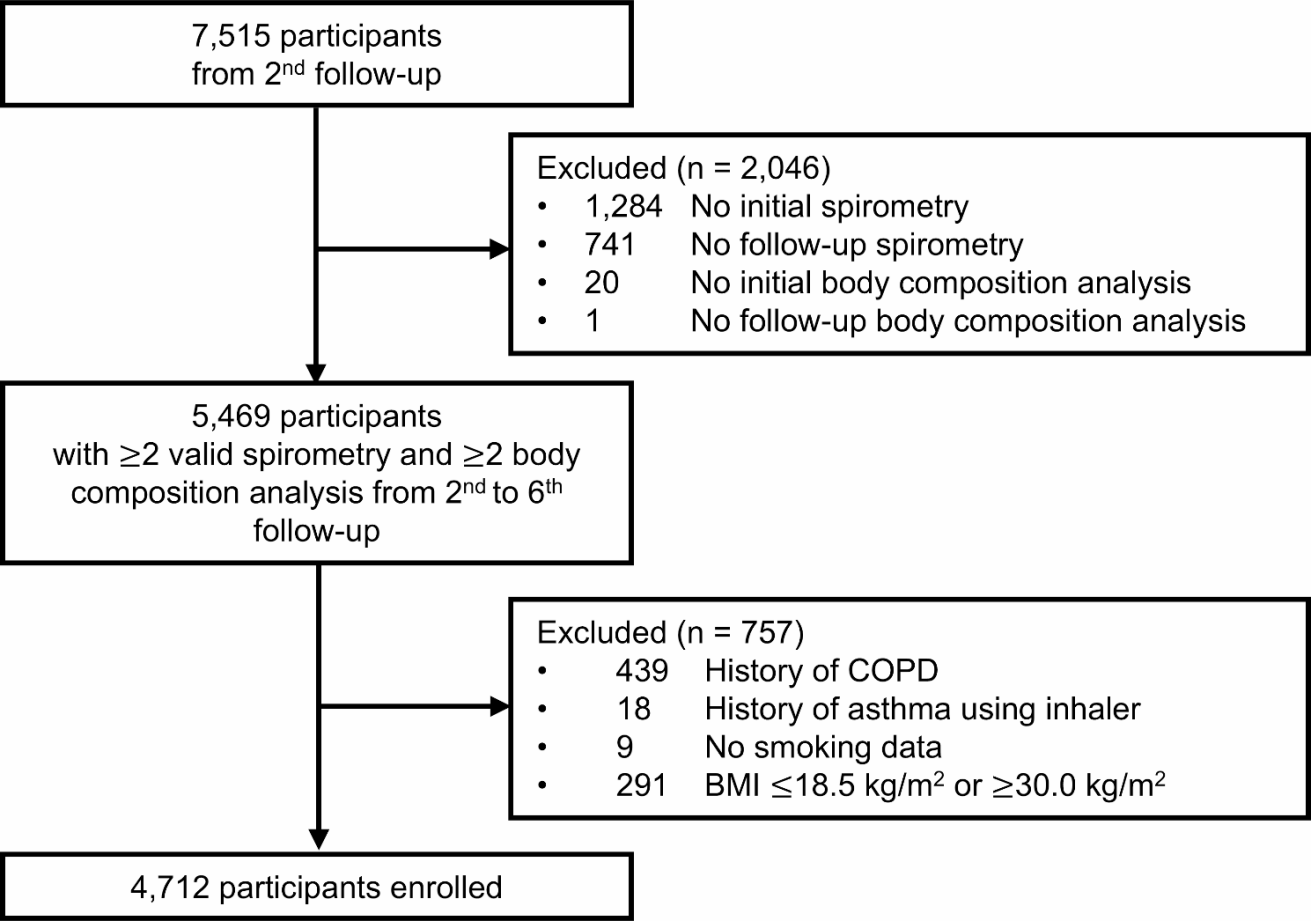
Flowchart of the participant selection process

Quality-controlled spirometry results were first obtained during the 2nd follow-up. Therefore, we used 7,515 participants from the 2nd follow-up (2005–2006) in this study, and they were tracked to the 6th follow-up (2013–2014). Those with valid spirometry and body composition analysis results from the initial assessment and at least another set of these results from the 3rd to the 6th follow-up were selected. Participants with a history of chronic lung disease or missing smoking data were excluded. Chronic lung disease was defined as a forced expiratory volume in 1s (FEV_1_)/forced vital capacity (FVC) ratio of less than 70% or the use of inhalers [17]. Due to the lack of handgrip strength and appendicular skeletal muscle mass data in the KoGES dataset, we could not exclude participants with sarcopenia. Instead, individuals with a body mass index (BMI) ≤18.5 kg/m^2^ (underweight) or ≥30.0 kg/m^2^ (severe obesity) were excluded [18, 19]. Consequently, 4,712 participants were enrolled in the study (Fig. 1).

### Spirometry

At baseline and during all the follow-up studies, lung function was evaluated through spirometry (Vmax- 2130, Sensor-Medics, Yorba Linda, CA). All tests were performed under the standardized protocols of the American Thoracic Society. Morris and Polgar’s equation was taken as a reference for normal lung function [20, 21].

### Body composition analysis

All anthropometric parameters were assessed using multi-frequency bioelectrical impedance analysis (InBody 3.0, InBody, Seoul, South Korea) [22]. An eight-point tactile electrode on the device measured impedance by analyzing the electrical characteristics of biological tissue. Previous research has established the validity of bioelectrical impedance analysis, exhibiting good agreement with dual-energy X-ray absorptiometry [23, 24].

The MF ratio was designated as the ratio of total body muscle mass (kg) to fat mass (kg). Muscle mass and fat mass divided by the square of height in meters were indicated as the muscle mass index and fat mass index, respectively [25, 26].

Long-term changes in individuals’ MF ratio were calculated through linear regression, and the participants were classified into three groups according to their MF ratio change slopes: “MF ratio-increased” with the upper 25% of MF ratio change slope, “MF ratio-decreased” with the lower 25% of MF change slope, and “MF ratio-stable” with between the upper 25% and lower 25% of MF change slope, including a zero-degree slope. This categorization was selected because the MF ratio lacks a standard cut-off point or normal range, and the cut-off points were determined based on the sample distribution. A three-group classification was adopted to offer a precise and meaningful representation of the long-term changes in the MF ratio.

### Statistical analyses

Pearson χ2 test and Fisher’s exact test compared categorical variables; these are presented as the numbers and their percentages (%). The Student’s t-test or the Mann–Whitney U test was used for continuous variables; these are presented as means ± standard deviation, or median (interquartile range [IQR], the first quartile (Q1)–the third quartile (Q3)). The association between the MF ratio and baseline lung function in men and women was derived through Pearson correlation analysis.

To show the overall longitudinal correlation between MF ratio change rate and lung function change rate, we calculated the change rates of MF ratio, FEV_1_, FVC, and FEV_1_/FVC through linear regression analyses.

Multiple linear mixed regression analyses were employed to clarify the longitudinal associations between MF ratio and lung function in each sex. We included only non-smoking women in the linear mixed regression analysis because a disproportionately low proportion (2.3%) of women had ever smoked. Age, height, residential area, exercise duration, and baseline lung function were adjusted. We calculated the weekly moderate-intensity exercise time at every follow-up and incorporated the change in exercise duration into the analyses. Smoking exposure (pack-years) was adjusted for men. In addition, we adjusted baseline lung function in the models because it might have affected the degree of lung function decline [27].

Generalized estimating equations (GEE) were used to clarify the odds ratio (OR) for the incidence of airflow obstruction according to the MF ratio. Airflow obstruction was defined as FEV_1_/FVC less than 70% [28]. GEE analyses require no missing data from the variables being applied. Therefore, we selected those who had undergone valid spirometry, body composition analysis, and exercise time evaluation at least 4 or 5 times during the follow-up periods. For the participants who had undergone these analyses 4 times, the missing values were replaced through multiple imputation, a popular generic method for examining data with missing value. Women who had smoked were excluded from the GEE analyses.

We used restricted maximum likelihood methods to calculate beta parameters, and the Wald test was used for the *P*-value. All statistical analyses were performed using R software v4.3.3 (The R Foundation for Statistical Computing, Vienna, Austria). The lme4 package was used for multiple linear mixed regression analyses, and the geepack package was used for GEE. Statistical significance was defined as a two-tailed *P*-value of < 0.05 for all analyses.

## Results

### Baseline characteristics

Table 1 shows the baseline characteristics and the follow-up data of the 4,712 participants (45.8% men) stratified by sex. Significant variation of data between men and women was demonstrated, including anthropometric and lung function indices. Men had a higher proportion of ever-smokers (73.4% vs. 2.4%, *P* < 0.001), higher muscle mass index (18.21 ± 1.32 kg/m^2^ vs. 15.92 ± 1.08 kg/m^2^, *P* < 0.001), and higher MF ratios (3.79 ± 1.19 vs. 2.23 ± 0.54, *P* < 0.001) than women, although fat mass index (5.22 ± 1.49 kg/m^2^ vs. 7.52 ± 1.74 kg/m^2^, *P* < 0.001) was lower in men. In terms of lung function, men had higher FVC (4.27 ± 0.63 L vs. 3.02 ± 0.50 L, *P* < 0.001) and FEV_1_ (3.37 ± 0.52 L vs. 2.46 ± 0.42 L, *P* < 0.001) than women, although FEV_1_/FVC (78.89 ± 4.78% vs. 81.41 ± 4.49%, *P* < 0.001) was lower. The median follow-up period was 8 years (IQR, 8–8), and the spirometry and body composition were measured for both, a median of 5 times (IQR, 4–5).

**Table 1:** Baseline characteristics and follow-up data of study participants

	Total (*N* = 4,712)	Men (*N* = 2,159)	Women (*N* = 2,553)	*P*-value
Age, years	53.9 ± 7.9	53.1 ± 7.4	54.6 ± 8.2	< 0.001
Height, cm	160.4 ± 8.6	167.4 ± 5.7	154.4 ± 5.5	< 0.001
Body mass index, kg/m^2^	24.4 ± 2.4	24.5 ± 2.4	24.4 ± 2.5	0.38
Smoking				< 0.001
Never	3070 (65.2)	576 (26.6)	2494 (97.7)	
Ex-smoker	872 (18.5)	854 (39.6)	22 (0.8)	
Current-smoker	770 (16.3)	729 (33.8)	41 (1.6)	
Smoking exposure, pack-year	8.64 ± 15.61	18.54 ± 18.51	0.27 ± 2.64	< 0.001
Residential area				< 0.001
Rural	1976 (41.94)	792 (36.7)	1184 (46.4)	
Urban	2736 (58.06)	1367 (63.3)	1369 (53.6)	
Exercise, hr/week	2.13 ± 3.56	2.42 ± 3.83	1.89 ± 3.29	< 0.001
Fat mass, kg	16.4 ± 4.4	14.6 ± 4.2	17.9 ± 4.1	< 0.001
Fat mass index, kg/m^2^	6.47 ± 1.99	5.22 ± 1.49	7.52 ± 1.74	< 0.001
Muscle mass, kg	44.0 ± 8.0	51.1 ± 5.5	38.0 ± 3.9	< 0.001
Muscle mass index, kg/m^2^	16.97 ± 1.65	18.21 ± 1.32	15.92 ± 1.08	< 0.001
Muscle/Fat ratio	2.95 ± 1.19	3.79 ± 1.19	2.23 ± 0.54	< 0.001
Lung function				
FVC, L	3.59 ± 0.84	4.27 ± 0.63	3.02 ± 0.50	< 0.001
FVC, % predicted	104.6 ± 12.5	102.0 ± 11.76	106.8 ± 12.7	< 0.001
FEV_1_, L	2.87 ± 0.65	3.37 ± 0.52	2.46 ± 0.42	< 0.001
FEV_1_, % predicted	113.0 ± 14.7	108.6 ± 13.1	116.8 ± 15.0	< 0.001
FEV_1_/FVC	80.26 ± 4.79	78.89 ± 4.78	81.41 ± 4.49	< 0.001
Measurements				
Spirometry, times	5 (4–5)	5 (4–5)	5 (4–5)	0.001
BIA, times	5 (4–5)	5 (4–5)	5 (4–5)	0.001
Exercise evaluation, times	5 (4–5)	5 (4–5)	5 (4–5)	0.003
Follow-up duration, year	8 (8–8)	8 (8–8)	8 (8–8)	0.008

*Data are presented as numbers (%), means ± standard deviation, or median (interquartile range) unless otherwise indicated

*FEV*_*1*_ forced expiratory volume in 1s, *FVC* forced vital capacity, *BIA* bioelectrical impedance analysis

### Cross-sectional association between MF ratio and baseline lung function

**Fig. 2:**
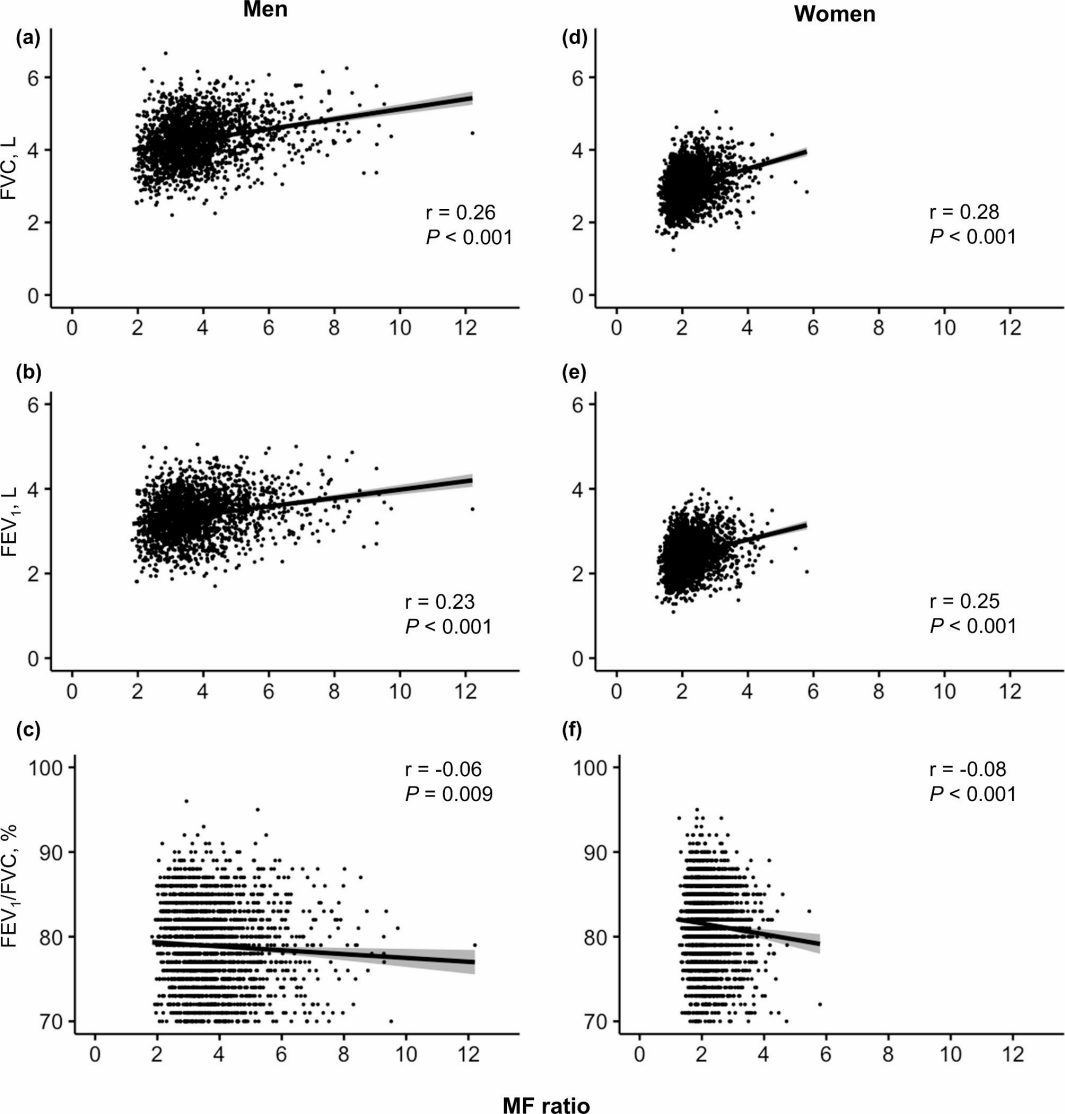
Association between MF ratio and baseline lung function in men and women. Pearson correlation between (**a**) FVC and MF ratio in men (*r* = 0.26, *P* < 0.001), (b) FEV1 and MF ratio in men (*r* = 0.23, *P* < 0.001), (c) FEV1/FVC and MF ratio in men (*r*=-0.06, *P* = 0.009), (d) FVC and MF ratio in women (*r* = 0.28, *P* < 0.001), (e) FEV1 and MF ratio in women (*r* = 0.25, *P* < 0.001), (f) FEV1/FVC and MF ratio in women (*r*=-0.08, *P* < 0.001) *FEV*_*1*_ forced expiratory volume in 1s, *FVC* forced vital capacity, *MF ratio* muscle-to-fat ratio

Since the results of body composition analyses differed between men and women, further analyses were performed in each sex. The associations between MF ratio and baseline lung function in men and women are shown in Fig. 2. MF ratio was positively related to FVC (men: *r* = 0.26, women: *r* = 0.28; all *P* < 0.001) and FEV_1_ (men: *r* = 0.23, women: *r* = 0.25; all *P* < 0.001) and negatively related to FEV_1_/FVC (men: *r*=-0.06, *P* = 0.009; women: *r*=-0.08, *P* < 0.001).

### Correlation between change rates of MF ratio and lung function

**Fig. 3:**
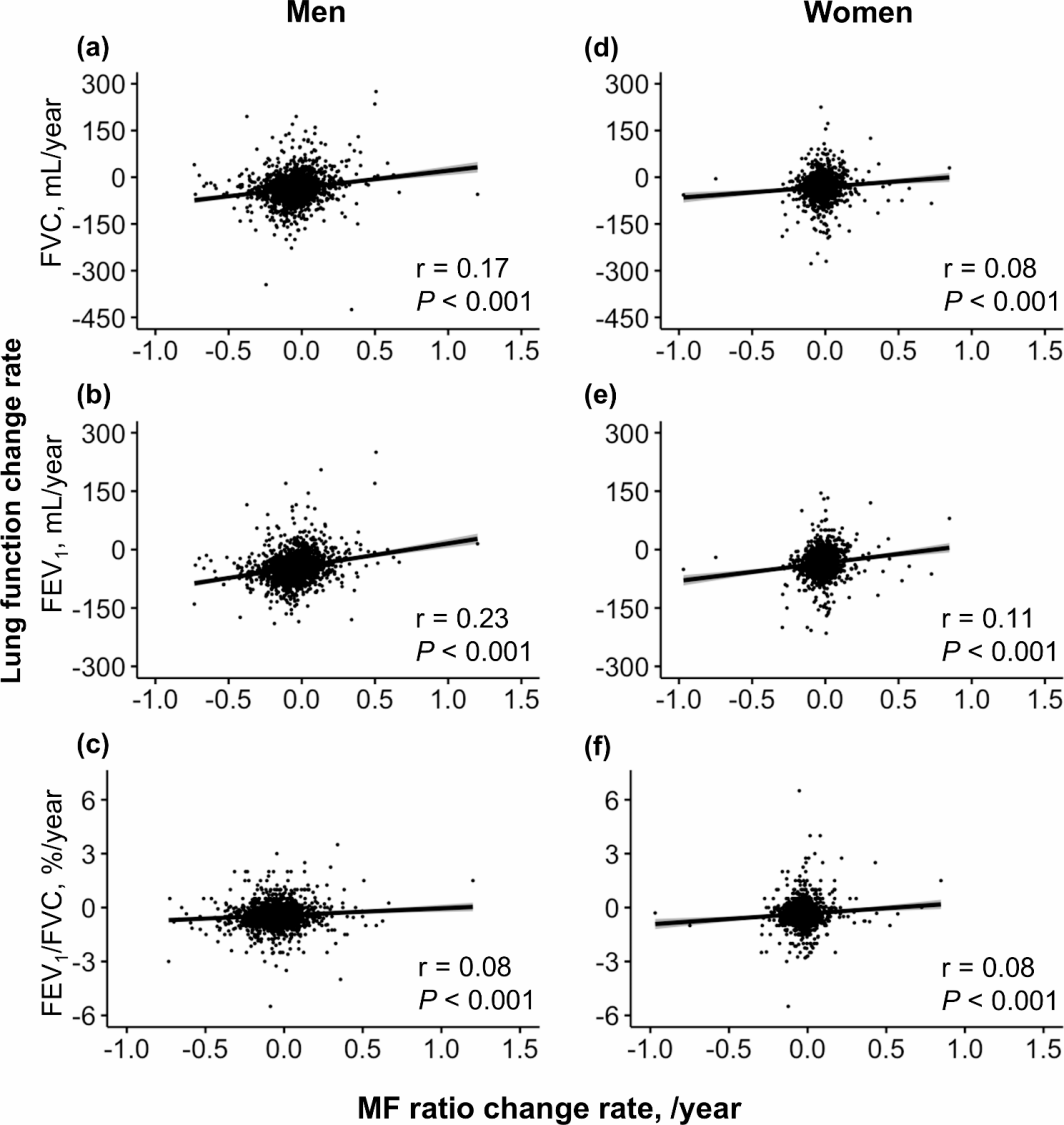
Correlation between MF ratio change rate and lung function change rate in men and women. (**a**) A change rate of FVC and an MF ratio change rate in men. (**b**) A change rate of FEV1 and an MF ratio change rate in men. (**c**) A change rate of FEV1/FVC and an MF ratio change rate in men. (**d**) A change rate of FVC and an MF ratio change rate in women. (**e**) A change rate of FEV1 and an MF ratio change rate in women. (**f**) A change rate of FEV1/FVC and an MF ratio change rate in women. An outlier in women with an FVC change rate of -850 mL/year, FEV1 change rate of -795 mL/year, and FEV1/FVC change rate of -6%/year was excluded *FEV*_*1*_ forced expiratory volume in 1s, *FVC* forced vital capacity, *MF ratio* muscle-to-fat ratio

The correlations between the change rates of the MF ratio and lung function are shown in Fig. 3. An increase in MF ratio change rate (/year) was significantly associated with an increase in lung function change rate in both men and women (all *P* < 0.001).

### Longitudinal association between MF ratio and lung function

Longitudinal changes in body composition and lung function between the initial assessment and 8 years of follow-up are presented in Supplementary Table 1. Multiple linear mixed regression analyses demonstrated the longitudinal association between the MF ratio and lung function (Table 2**).** With an increase in the MF ratio by 1, the FVC in men increased by 43.9 mL, FEV_1_ by 37.6 mL, and FEV_1_/FVC by 0.320% (all *P* < 0.001). Moreover, with an increase in the MF ratio by 1, the FVC in non-smoking women increased by 55.8 mL, FEV_1_ by 44.3 mL, and FEV_1_/FVC by 0.265% (all *P* < 0.001).

**Table 2:** Multiple linear mixed regression analysis of long-term associations between MF ratio and lung function in men and non-smoking women

	FVC, mL				FEV_1_, mL				FEV_1_/FVC, %			
Men (*N* = 2,159)	Estimate	Std Err	*P*-value	*95% CI*	Estimate	Std Err	*P*-value	*95% CI*	Estimate	Std Err	*P*-value	*95% CI*
MF ratio	43.9	2.4	< 0.001	39.0, 48.7	37.6	2.2	< 0.001	33.0, 42.1	0.320	0.034	< 0.001	0.251, 0.388
Age, years	–2.4	0.5	< 0.001	-3.4, -1.5	–2.3	0.4	< 0.001	-3.1, -1.5	-0.005	0.007	0.454	-0.018, 0.008
Height, cm	2.2	0.7	< 0.001	0.9, 3.5	0.9	0.6	0.098	-0.2, 2.0	–	–	–	−
Smoking exposure, pack-year	0.0	0.2	0.858	-0.3, 0.4	–0.2	0.1	0.197	-0.5, 0.1	-0.004	0.002	0.094	-0.009, 0.001
Area – Urban	–66.5	6.7	< 0.001	-79.7, -53.4	–16.5	5.9	0.005	-28.1, -5.0	0.999	0.099	< 0.001	0.805, 1.192
Exercise, hr/week	1.0	0.6	0.099	-0.2, 2.3	0.7	0.6	0.226	-0.5, 1.9	-0.005	0.009	0.578	-0.023, 0.013
Baseline lung function	905.2	6.4	< 0.001	892.7, 917.8	901.6	6.8	< 0.001	888.2, 915.0	0.939	0.010	< 0.001	0.920, 0.958
**Women, non-smoker (** *N* ** = 2,494)**												
MF ratio	55.8	3.3	< 0.001	49.1, 62.4	44.3	3.0	< 0.001	38.2, 50.4	0.265	0.053	< 0.001	0.160, 0.370
Age, years	–1.7	0.3	< 0.001	-2.3, -1.1	–1.7	0.3	< 0.001	-2.3, -1.2	-0.021	0.005	< 0.001	-0.031, -0.012
Height, cm	2.0	0.5	< 0.001	1.0, 2.9	0.5	0.4	0.192	-0.3, 1.3	–	–	–	−
Area – Urban	–71.7	4.7	< 0.001	-80.9, -62.6	–42.7	4.0	< 0.001	-50.5, -34.8	0.592	0.077	< 0.001	0.442, 0.743
Exercise, hr/week	2.4	0.5	< 0.001	1.3, 3.4	1.4	0.5	0.006	0.4, 2.4	-0.011	0.009	0.201	-0.028, 0.006
Baseline lung function	890.4	6.2	< 0.001	878.2, 902.6	896.2	6.3	< 0.001	883.8, 908.6	0.914	0.008	< 0.001	0.898, 0.930

*MF ratio* muscle-to-fat ratio, *CI* confidence interval

**Fig. 4:**
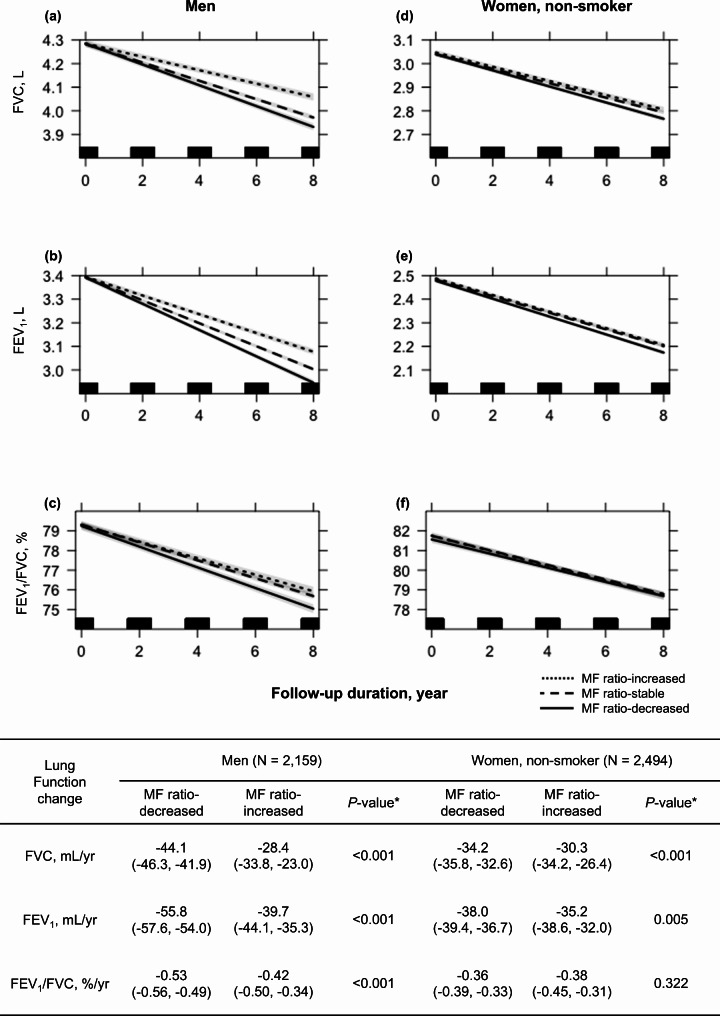
Multiple linear mixed regression analysis for lung function decline according to MF ratio change. Lung function decline is compared between the MF ratio-decreased, -stable, and -increased groups in men and non-smoking women, respectively. MF ratio-decreased group is used as the reference group. The decline in (**a**) FVC in men, (**b**) FEV1 in men, (**c**) FEV1/FVC in men, (**d**) FVC in non-smoking women, (**e**) FEV1 in non-smoking women, (**f**) FEV1/FVC in non-smoking women. Individual changes in MF ratio during follow-up are calculated using linear regression analysis. Participants with a lower 25% MF ratio change are assigned to the MF ratio-decreased group, those between lower 25% and upper 25% are assigned to the MF ratio-stable group, which includes a zero-degree slope, and those with an upper 25% MF ratio change are assigned to the MF ratio-increased group. Results are adjusted for age, height, residential area, weekly moderate-intensity exercise duration, follow-up duration, and initial lung function. For men, smoking exposure (pack-years) is also adjusted. The gray shadow and numbers in parentheses represent 95% confidence intervals. *P-value of MF ratio-increased group compared with MF ratio-decreased group *FEV*_*1*_ forced expiratory volume in 1s, *FVC* forced vital capacity, *MF ratio* muscle-to-fat ratio

According to individual changes in the MF ratio during the follow-up period, the patients were divided into three groups: MF ratio increased, -stable (including zero-degree slope), and -decreased. The longitudinal associations between lung function and the three groups are demonstrated in Fig. 4. In comparison with the MF ratio-decreased group, the MF ratio-increased group showed attenuation of lung function decline for both men (FVC: -44.1 mL/year vs. -28.4 mL/year, FEV_1_: -55.8 mL/year vs. -39.7 mL/year, FEV_1_/FVC: -0.53%/year vs. -0.42%/year, all *P* < 0.001) and non-smoking women (FVC: -34.2 mL/year vs. -30.3 mL/year, *P* < 0.001; FEV_1_: -38.0 mL/year vs. -35.2 mL/year, *P* = 0.005).

### Relationship between MF ratio and airflow obstruction

Table 3 demonstrates the relationship between the MF ratio level and airflow obstruction in men and non-smoking women. During the follow-up, 369 (20.1%) out of 1,837 men and 154 (7.0%) out of 2,202 non-smoking women developed airflow obstruction. As the MF ratio increased, the incidence of airflow obstruction showed a significant decrease in both men (OR: 0.77, 95% CI: 0.68–0.87, *P* < 0.001) and non-smoking women (OR: 0.85, 95% CI: 0.74–0.97, *P* = 0.014).

**Table 3:** Odds ratio for incidence of airflow obstruction according to MF ratio in men and non-smoking women

	Men (*N* = 1,837)		Women, non-smoker (*N* = 2,202)	
	OR (95% CI)	*P*-value	OR (95% CI)	*P*-value
MF ratio	0.77 (0.68, 0.87)	< 0.001	0.85 (0.74, 0.97)	0.014
Age, years	1.05 (1.03, 1.06)	< 0.001	1.05 (1.02, 1.07)	< 0.001
Smoking exposure, pack-year	1.01 (1.01, 1.02)	< 0.001	–	–
Area – Urban	2.19 (1.71, 2.80)	< 0.001	1.90 (1.29, 2.80)	0.001
Exercise, hr/week	1.00 (0.98, 1.02)	0.659	0.99 (0.94, 1.03)	0.589

*MF ratio* muscle-to-fat ratio, *CI* confidence interval, *OR* odds ratio

## Discussion

We demonstrated that changes in the MF ratio are associated with lung function decline in the middle-aged general population. A cross-sectional analysis found that the participants’ baseline FVC and FEV_1_ were positively related to the MF ratio. Longitudinally, there was a positive correlation between lung function and the MF ratio. Those with an increase in the MF ratio had an attenuated decline in FVC and FEV_1_ than those with a decrease in the MF ratio over time. Moreover, an increase in the MF ratio decreased the risk of airflow obstruction.

Several cross-sectional studies assessed the association between body composition and lung function [10, 11, 15, 29]. Both sarcopenia and obesity were independent risk factors for worsened lung function in male patients with COPD [11]. Individuals without any known lung diseases also had a positive correlation between skeletal muscle index (skeletal muscle mass, kg, divided by square of height, m^2^) and FVC, FEV_1_, and peak expiratory flow [10]. A few longitudinal studies have investigated the influence of body composition on lung function, employing adiposity, especially the increase in abdominal fat indices [27, 30]. However, few studies have longitudinally investigated both muscle and fat mass with lung function in the general population. A retrospective analysis of health check-up data of individuals by Park et al. [9] showed that gain of muscle mass with loss of fat mass was related to decline in FEV_1_. In this study, we demonstrated the long-term relationship between the MF ratio and lung function using a large number of individuals randomly selected from the general community.

Several parameters can reflect body composition. This study used the MF ratio to represent overall muscle and fat mass. The reasons were as follows. First, investigating the MF ratio, rather than BMI, allowed better discrimination between individuals with different body compositions. Individuals with the same BMI may not have the same muscle and fat mass. Studies have demonstrated that BMI is imperfect when evaluating body composition, including obesity [31]. Therefore, considering both muscle and fat composition can represent our body composition more precisely. Second, by combining both muscle and fat mass into one variable, the MF ratio could demonstrate the body composition comprehensively and simply, without additional categorization, to clarify the impact of muscle and fat on each other. Therefore, a more holistic view of body composition improved the ability to capture its significant effect on lung function [32]. Third, as the MF ratio is related to various diseases, we believe examining the change in the MF ratio had greater scientific significance. The MF ratio is an index of sarcopenic obesity and indicates the metabolic and inflammatory status, which predicts insulin resistance and metabolic syndrome [33, 34]. Moreover, the MF ratio is related to the development of chronic kidney disease in individuals with normal renal function [33, 35].

The MF ratio affects lung function through mechanical and inflammatory pathways. Gaining fat mass increases carbon dioxide production and oxygen consumption, which stiffens the respiratory system. Obesity can also lower lung volumes, such as closing capacity and functional residual capacity, potentially leading to hypoxemia and increasing the effort of breathing [36, 37]. Conversely, loss of muscle mass further reduces lung volumes and increases lung restriction [10]. As muscle mass declines, inflammatory mediators such as serum C-reactive protein increase, which may compromise lung flexibility and expansion [38, 39].

In our cohort, individuals with an increased MF ratio likely experienced reduced fat mass and preserved or increased muscle mass, which positively affected lung function. Reduced fat mass improves lung compliance and decreases respiratory system stiffness, whereas increased muscle mass enhances respiratory muscle strength and breathing efficiency. These changes collectively explain the observed associations, where a higher MF ratio correlated with better lung function measures, reinforcing its role as a key determinant of lung function over time.

This study has strengths in that it excluded those who had chronic lung diseases and those who were underweight or severely obese. Moreover, because we prospectively followed up the participants for 8 years, we explored the impact of the MF ratio on lung function more accurately and adequately. Nonetheless, this study has some limitations. First, airflow obstruction was defined solely based on pulmonary function test without considering symptoms. Second, underdiagnosed airflow obstruction may result from using the definition of FEV_1_/FVC less than 70%. The physiologically appropriate cut-off for airflow obstruction may be the lower limit of normal, which is determined statistically by a reference population’s lower fifth percentile [40]. However, evidence indicates no significant differences in comorbidities or prognosis between patients classified by the fixed ratio and the lower limit of normal [41]. Therefore, we adopted the fixed ratio (FEV_1_/FVC < 70%) as a definition of airflow obstruction in alignment with both international guidelines and its established relevance to our study population [42, 43]. Third, the causality between the MF ratio and lung function remains unclear. Whether an increase in the MF ratio results in less decline in lung function or whether worsening of lung function causes a decrease in the MF ratio cannot be fully demonstrated. Fourth, the adjusted models did not account for dietary habits, which may have influenced the relationship between body composition and lung function. Although previous studies using KoGES data have explored various dietary factors and their association with lung function [44, 45], incorporating these diverse variables into a single statistical model was deemed impractical and potentially confounding. Finally, lung function assessments were done exclusively through pre-bronchodilator spirometry, without including post-bronchodilator spirometry or imaging studies. However, by conducting spirometry under strict quality-controlled conditions, we ensured the reliability of our lung function assessments.

In conclusion, longitudinal changes in the MF ratio positively correlate with lung function. Furthermore, the probability of airflow obstruction decreases as the MF ratio increases. Therefore, individuals with altered body composition, especially of the MF ratio, should be monitored for lung function deterioration and the possibility of developing obstructive lung diseases. Further studies are necessary to investigate whether increasing the MF ratio can help prevent lung function decline and development of chronic obstructive pulmonary disease.

## Electronic supplementary material

Below is the link to the electronic supplementary material.

Supplementary Material 1

## Data Availability

The data used in this study are publicly available from Korean National Institute of Health, Clinical and Omics Data Archive (CODA) at https://coda.nih.go.kr/.

## Abbreviations

COPD: Chronic obstructive pulmonary disease
MF: Muscle-to-fat
BMI: Body mass index
FEV_1_: Forced expiratory volume in 1s
FVC: Forced vital capacity
GEE: Generalized estimating equations
OR: Odds ratio
BIA: Bioelectrical impedance analysis
CI: Confidence interval
