# Supplementary material for: Changes in muscle-to-fat ratio are associated with lung function decline and airflow obstruction in the general population
Source: Respir Res. 2024 Dec 26;25:444. doi: 10.1186/s12931-024-03081-w (PMC11673890; doi:10.1186/s12931-024-03081-w)
Supplement: Supplementary file 1 — Supplementary Material 1 [file 12931_2024_3081_MOESM1_ESM.docx]

**Supplementary Table 1**. Changes of body composition, lung function parameters, and exercise duration through follow-up.

| Parameters | Men (N=2,159) | | Women (N=2,553) | |
| --- | --- | --- | --- | --- |
|  | Initial | after 8 years | Initial | after 8 years |
| Fat mass, kg | 14.62 ± 4.19 | 16.27 ± 4.87 | 17.88 ± 4.08 | 19.42 ± 4.80 |
| Muscle mass, kg | 51.12 ± 5.50 | 49.39 ± 5.49 | 38.03 ± 3.91 | 36.09 ± 3.96 |
| Muscle/Fat ratio | 3.79 ± 1.19 | 3.31 ± 1.07 | 2.23 ± 0.54 | 1.97 ± 0.53 |
| FVC, L | 4.27 ± 0.63 | 3.99 ± 0.61 | 3.02 ± 0.50 | 2.80 ± 0.46 |
| FVC, % predicted | 102.0 ± 11.76 | 100.51 ± 12.27 | 106.8 ± 12.7 | 106.48 ± 13.67 |
| FEV_1_, L | 3.37 ± 0.52 | 3.01 ± 0.49 | 2.46 ± 0.42 | 2.21 ± 0.38 |
| FEV_1_, % predicted | 108.6 ± 13.1 | 105.86 ± 14.01 | 116.8 ± 15.0 | 115.83 ± 17.01 |
| FEV_1_/FVC | 78.89 ± 4.78 | 75.38 ± 5.67 | 81.41 ± 4.49 | 78.78 ± 5.07 |
| Exercise, hr/week | 2.42 ± 3.83 | 2.60 ± 3.81 | 1.89 ± 3.29 | 1.72 ± 3.03 |

*FEV_1_* forced expiratory volume in 1s, *FVC* forced vital capacity
